# Genetic micro-epidemiology of malaria in Papua Indonesia: Extensive *P*. *vivax* diversity and a distinct subpopulation of asymptomatic *P*. *falciparum* infections

**DOI:** 10.1371/journal.pone.0177445

**Published:** 2017-05-12

**Authors:** Zuleima Pava, Rintis Noviyanti, Irene Handayuni, Hidayat Trimarsanto, Leily Trianty, Faustina H. Burdam, Enny Kenangalem, Retno A. S. Utami, Yusrifar K. Tirta, Farah Coutrier, Jeanne R. Poespoprodjo, Ric N. Price, Jutta Marfurt, Sarah Auburn

**Affiliations:** 1 Global and Tropical Health Division, Menzies School of Health Research, Charles Darwin University, Darwin, Northern Territory, Australia; 2 Malaria Pathogenesis Unit, Eijkman Institute for Molecular Biology, Jakarta, Indonesia; 3 Bioinformatics Laboratory, Eijkman Institute for Molecular Biology, Jakarta, Indonesia; 4 Agency for Assessment and Application of Technology, Jakarta, Indonesia; 5 Mimika District Health Authority, Timika, Papua, Indonesia; 6 Timika Malaria Research Programme, Papuan Health and Community Development Foundation, Timika, Papua, Indonesia; 7 Pediatric Research Office, Department of Child Health, Faculty of Medicine, Universitas Gadjah Mada, Yogyakarta, Indonesia; 8 Centre for Tropical Medicine, Nuffield Department of Clinical Medicine, University of Oxford, Oxford, United Kingdom; Johns Hopkins Bloomberg School of Public Health, UNITED STATES

## Abstract

**Background:**

Genetic analyses of *Plasmodium* have potential to inform on transmission dynamics, but few studies have evaluated this on a local spatial scale. We used microsatellite genotyping to characterise the micro-epidemiology of *P*. *vivax* and *P*. *falciparum* diversity to inform malaria control strategies in Timika, Papua Indonesia.

**Methods:**

Genotyping was undertaken on 713 sympatric *P*. *falciparum* and *P*. *vivax* isolates from a cross-sectional household survey and clinical studies conducted in Timika. Standard population genetic measures were applied, and the data was compared to published data from Kalimantan, Bangka, Sumba and West Timor.

**Results:**

Higher diversity (*H*_*E*_ = 0.847 vs 0.625; *p* = 0.017) and polyclonality (46.2% vs 16.5%, *p*<0.001) were observed in *P*. *vivax* versus *P*. *falciparum*. Distinct *P*. *falciparum* substructure was observed, with two subpopulations, K1 and K2. K1 was comprised solely of asymptomatic infections and displayed greater relatedness to isolates from Sumba than to K2, possibly reflecting imported infections.

**Conclusions:**

The results demonstrate the greater refractoriness of *P*. *vivax* versus *P*. *falciparum* to control measures, and risk of distinct parasite subpopulations persisting in the community undetected by passive surveillance. These findings highlight the need for complimentary new surveillance strategies to identify transmission patterns that cannot be detected with traditional malariometric methods.

## Introduction

Indonesia has one of the highest burdens of malaria in Southeast Asia. Despite recent progress in reducing case incidence, more than a quarter of a million clinical cases were reported in 2013 with 230 million people at risk of infection [1]. The high heterogeneity of malaria epidemiology across approximately 6,000 inhabited islands is a huge challenge to aspirations for malaria control and its ultimate elimination. Across the archipelago, marked differences are observed in malaria incidence, *Anopheles* vector distributions, and ethnic and socio-economic features [2]. There is a continual flux in parasite populations in response to the changing environment, combined with a highly mobile human population, resulting in a dynamic malaria epidemiology at any site.

Further complicating the picture, all five species of *Plasmodium* that commonly infect humans are present in Indonesia [2]. Differences in the respective transmission of these species confound prioritization of malaria control activities. Although *P*. *falciparum* has historically been the predominant species, in recent years the proportion of *P*. *vivax* cases has risen [3]. This trend is especially concerning due to the occurrence of high-grade multidrug-resistance in the historically neglected *P*. *vivax* species, particularly in Papua Province [4, 5].

Population genetic approaches can define *P*. *vivax* and *P*. *falciparum* diversity and structure, and inform associated patterns of transmission intensity, foci and stability, thus complementing traditional malariometric surveillance [6–9]. A recent nationwide Indonesian study applied parasite genotyping to contrast patterns of *P*. *vivax* and *P*. *falciparum* transmission between four sites in Indonesia with varying endemicity, highlighting regional heterogeneity within and between species [10]. However, control and elimination activities are generally implemented at district-level spatial scales [11], and little is known about the heterogeneity in parasite populations at this administrative level.

The aim of this study was to describe the population diversity and structure of co-endemic *P*. *falciparum* and *P*. *vivax* circulating in Timika, the capital of Mimika district, Papua Province. The region is an epicentre for antimalarial resistance [4, 5] and thus, with the threat of resistance arising against artemisinin-based combination therapies (ACTs), effective strategies are needed to interrupt local malaria transmission as quickly and cost-effectively as possible. We describe the implications of our findings in regards to the local transmission dynamics and appropriate malaria control and elimination strategies.

## Materials and methods

### Study site and sampling

The study was conducted in Timika, Papua Province in eastern Indonesia, which has the greatest burden of malaria in the country. Timika is located within Mimika Baru, one of the largest sub-districts in Mimika district. The population of 202,350 people is increasing in size by an estimated 16% per year, mainly due to economic migration, and is composed of different ethnic groups including Highland Papuans, Lowland Papuans, and Non-Papuans. Unstable malaria transmission occurs mainly in the lowland areas, where approximately 90% of the population lives. In 2013, malaria prevalence by microscopy was estimated to be 12.2% (5.7% due to *P*. *vivax* and 5.2% due to *P*. *falciparum*) [2, 3].

The Rumah Sakit Mitra Masyarakat (RSMM) is the main hospital in the district, serving a population of approximately 150,000 people. Located in the lowland area, RSMM provides medical healthcare to approximately 300 patients per day. Since 2006, local antimalarial guidelines have recommended that all patients with uncomplicated malaria due to any *Plasmodium* species are treated with dihydroartemisinin-piperaquine (DHP).

Parasite DNA was collected within the framework of two studies. The first was an ongoing ex-vivo drug susceptibility surveillance study conducted between March 2011 and August 2014. The second was a cross-sectional study conducted between April and July 2013. Patient and parasitological details were recorded for each case. For the cross-sectional study, GPS coordinates were recorded of the residence of all individuals assessed. The majority of houses were located in one of 16 villages, within the 3 subdistricts Mimika Baru, Mimika Timur, and Kuala Kencana. Geospatial mapping of residences with *Plasmodium* cases was performed using ArcGIS software (version 10.2.1).

### DNA extraction and species confirmation

For parasitaemia detected by microscopy, species and parasite density were determined by examination of Giemsa-stained thick blood films. Genomic DNA (gDNA) was extracted from 50 μL packed red blood cell (RBC) pellets using the QIAamp 96 DNA Blood Kit (Qiagen) for the cross-sectional study and from 2 mL of packed RBCs using the QIAamp DNA Mini Kit (Qiagen) for the ex-vivo surveillance study cases. Confirmation of *Plasmodium* species was undertaken using a nested PCR protocol with 2 μL gDNA template in duplicate [12]. *P*. *falciparum*, *P*. *vivax*, *P*. *malariae*, and *P*. *ovale* small-subunit rRNA DNA clones (MRA-177, MRA-178, MRA-179, and MRA-180; ATCC, Manassas, VA) were used as positive controls.

### Genotyping and data analysis

*P*. *falciparum* and *P*. *vivax* genotyping was performed using short tandem repeat (STR) markers. Nine STR markers (ARAII, PfPK2, Poly-alpha, TA1, TA42, TA60, TA81, TA87 and TA109) were amplified in *P*. *falciparum* infections using previously described methods [13]. For *P*. *vivax*, nine STR markers that have been defined as a consensus panel within the Asia Pacific Malaria Elimination Network (APMEN) (*Pv3*.*27*, MS16, MS1, MS5, MS10, MS12, MS20 and msp1F3) were amplified using the APMEN protocol [14–16]. Capillary electrophoresis on an ABI 3100 Genetic Analyser with GeneScan LIZ-600 (Applied Biosystems) was used to separate the fluorescently labelled PCR products. The resulting electrophoretograms were analysed using GeneMapper version 4.0 software (Applied Biosystems) and verified manually. To reduce artefact peaks, an arbitrary fluorescent intensity threshold of 50 RFU was applied, and minor alleles were only scored when their height was at least 33% of the main allele’s height. Only samples with a minimum of 50% successful genotype calls across the loci investigated were included in downstream population genetic analyses.

Samples with more than one allele at any locus were defined as polyclonal infections. The multiplicity of infection (MOI) for each sample was defined as the maximum number of alleles at any locus. Population diversity was measured using the expected heterozygosity (*H*_E_), population differentiation was measured using the pairwise *F*_ST_ metric. Both *H*_E_ and *F*_ST_ were calculated using Arlequin software (version 3.5) [17, 18]. For *P*. *vivax*, measures of the *H*_E_, genetic differentiation and LD (described below) were undertaken on the full marker set and on a sub-set of five markers (MS1, MS5, MS10, MS12, MS20) defined as exhibiting balanced diversity in a recent study [19].

STRUCTURE software version 2.3.3 was used to assess population structure [20]. The simulation was run using 20 replicates, with 100,000 burn-in and 100,000 post burn-in iterations for each estimate of *K* (number of sub-populations), ranging from 1–10. The model parameters included admixture with correlated allele frequencies. The most probable *K* was derived by applying the *delta K* method [21]. STRUCTURE results were displayed using bar plots prepared with Distruct software version 1.1. [22]. Multi-locus genotypes (MLGs) were reconstructed from the predominant allele at each locus in isolates with no missing data. Using the MLGs, multi-locus linkage disequilibrium (LD) was measured by the standardised index of association (*I*_A_^S^) using the web-based LIAN 3.5 software [23]. The significance of the estimates was assessed using 10,000 permutations of the data. Given that the measures of LD and other MLG-based analyses could be affected by incorrect MLG reconstruction in complex polyclonal infections, the analysis was also performed using low complexity infections (maximum of 1 multi-allelic locus) with each unique MLG represented just once. The proportion of alleles shared between MLGs (ps) was calculated using 1-ps as a measure of genetic relatedness [24]. Unrooted neighbour-joining trees were generated with the ape package in R [25]. Mantel’s r-test was used to assess the correlation between genetic and either temporal or spatial distances using the ade4 package in R [26].

### Statistical tests

Statistical analysis was conducted using SPSS v.20.0 for windows software (IBM SPSS Statistics), with a significance threshold of 0.05. Data on patient gender, ethnicity and proportion of polyclonal infections was compared between subgroups using Pearson’s Chi-square test. The significance of difference between patient age, parasite density, MOI and *H*_E_ was undertaken using the Mann-Whitney U test and Kruskal-Wallis test.

### Ethics

Ethical approval for this study was obtained from the Eijkman Institute Research Ethics Commission, Eijkman Institute for Molecular Biology, Jakarta, Indonesia (EIREC no. 47/2010 and EIREC no. 67/2013), Faculty of Medicine, University of Gadjah Mada (Ref: KE/FK/763/EC), and the Human Research Ethics Committee of the Northern Territory Department of Health & Families and Menzies School of Health Research, Darwin, Australia (Ref: HREC-2010-1434).

## Results

### Sampling and data quality

Between March 2011 and August 2014, 108 *P*. *vivax* and 94 *P*. *falciparum* cases were collected from the surveillance study, with genotyping successfully completed in 96% (104) of *P*. *vivax* and all *P*. *falciparum* cases. The cross-sectional household survey, undertaken between April and July 2013, assessed 2,476 participants by blood film examination and PCR analysis, of whom 877 (35.4%) had peripheral parasitaemia detected by PCR. *P*. *vivax* and *P*. *falciparum* accounted for 749 (85.4%) malaria cases. Genotyping was successful in 60% (240/397) of *P*. *vivax* and 80% (280/352) of *P*. *falciparum* cases. A total of 73 (30.4%) *P*. *vivax* and 21 (7.5%) *P*. *falciparum* samples failed to amplify any of the markers; all of these had submicroscopic parasitaemia.

Demographic and parasitological details on the individuals with *P*. *falciparum* or *P*. *vivax* parasitaemia are presented in Table 1. There were no significant differences in individual’s age, gender, proportion of patent or symptomatic infections, or parasite density between the species. The geographical distribution of *P*. *vivax* and *P*. *falciparum* cases did not reveal any local high prevalence clusters for either species in the cross-sectional study S1 Fig.

**Table 1:** Demographic information.

Species	Age^1^, *n* (%)			Male patients, *n* (%)	Subpatent^2^, *n* (%)	Symptomatic infections, *n* (%)	Parasite density, median (range)
	<5	5–15 y	>15				
***P*. *falciparum***	27 (7.4)	87 (23.7)	253 (68.9)	157 (42.8)	159 (43.1)	105 (28.6)	5,475 (28–361,728)
***P*. *vivax***	45 (13.2)	92 (27.0)	204 (59.8)	147 (43.0)	161 (46.8)	107 (31.3)	6,735 (38–120,576)

Superscript indicates number of individuals with missing data.

The diversity and genotyping success rate for each of the *P*. *falciparum* and *P*. *vivax* markers are summarised in S1 Table. Although diversity was moderately low in two *P*. *falciparum* markers (TA42 *H*_E_ = 0.315 and TA109 *H*_E_ = 0.176), all markers exhibited a minimum 5% minor allele frequency. With the exception of TA42 (20.6%) in *P*. *falciparum* populations, all other markers exhibited >80% genotyping success in both species. The MS8 assay presented artefacts that affected the reliability of the allele calling and therefore, this locus was excluded from further analysis.

### Higher genetic diversity in co-endemic *P*. *vivax* versus *P*. *falciparum*

*P*. *vivax* exhibited higher genetic diversity than *P*. *falciparum* (*H*_E_ = 0.847 vs 0.625; *p* = 0.017) and a higher proportion of polyclonal infections (46.2% vs 16.5%, *p*<0.001). The mean MOI was higher for *P*. *vivax* (1.6) than *P*. *falciparum* (1.2) (*p*<0.001) (Table 2). In contrast to *P*. *falciparum*, there was no or low LD detected for *P*. *vivax* (Table 3). Analysis of all 8 *P*. *vivax* markers revealed no significant LD (*I*_*A*_^*S*^ = -0.0002, *p*>0.05), even after excluding complex infections (*I*_*A*_^*S*^ = -0.0008, *p*>0.05). There was no evidence of LD after restricting to the 5 balanced markers (*I*_*A*_^*S*^ = 0.0036, *p* > 0.05), although LD became significant after removing complex samples (*I*_*A*_^*S*^ = 0.006, 0.01≤*p*< 0.05; Table 3) [19]. Further MLG-based analyses restricted to the low complexity samples corroborated that the polyclonal samples were not affecting MLG reconstruction and subsequent results. Significant LD was observed in *P*. *falciparum*, with consistently high *I*_*A*_^*S*^ values varying from 0.115–0.103 (all *p*<0.001). LD analysis using the unique haplotypes found no evidence of epidemic transmission in either species (Table 3).

**Table 2:** Within-host and population diversity.

Species and subgroup (K)	*n*	MOI, mean (range)	Polyclonality, %	*H*_E_, mean ± SD
*P*. *vivax*	344	1.6 (1–4)	46.2	0.849 ± 0.07
*P*. *falciparum*	369	1.2 (1–3)	16.5	0.656 ± 0.20
*P*. *falciparum K1*	39	1.5 (1–3)	43.6	0.587 ± 0.27
*P*. *falciparum K2*	236	1.1 (1–2)	14.4	0.556 ± 0.29

**Table 3:** Linkage disequilibrium.

Species and subgroup (K)	All infections, *n*	*I*_*A*_^*S*^	Low complexity infections, *n*	*I*_*A*_^*S*^	Unique MLGs, *n*	*I*_*A*_^*S*^
*P*. *vivax*^8^	223	-0.0002^NS^	145	-0.0008^NS^	223	-0.0002^NS^
*P*. *vivax*^5^	250	0.0036^NS^	186	0.006*	245	0.0021^NS^
*P*. *falciparum*	262	0.1150**	237	0.1136**	188	0.1031**
*P*. *falciparum K1*	28	0.0405**	24	0.0249**	23	0.0121^NS^
*P*. *falciparum K2*	234	0.0323**	213	0.0334**	165	0.0133**

Only samples with no missing data were included in the analyses. All nine loci were used in *P*. *falciparum*. Superscript indicates the number of loci used for the analysis in the *P*. *vivax* populations.

* *p* <0.05

** *p*< 0.01

*** *p*<0.001

NS = Not significant

### Low level sharing of identical *P*. *falciparum* infections amongst household members

Thirty three *P*. *falciparum* MLGs (106 infections) were observed in more than one individual (range 2–13). Only 22 MLGs (68 cases) had GPS metadata available. Amongst these, 3 pairs of infections (8.8% (6/68)), were shared between members of the same household. Seven pairs of infections (35.3%, (24/68)) were less than 1 Km apart. The median distance between infections was 5.3 Km (range 2 meters to 19.5 Km) (S2 Fig).

### Distinct subpopulations *of P*. *falciparum* in Timika

**Fig 1:**
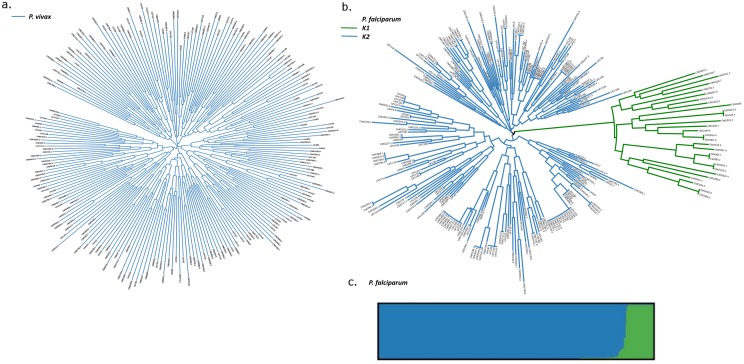
Neighbour-joining and STRUCTURE plots illustrating the genetic diversity and structure in the *P*. *vivax* and *P*. *falciparum* populations in Timika. Panels A) and B) present unrooted neighbour-joining trees illustrating the genetic relatedness amongst *P*. *vivax* and *P*. *falciparum* isolates, respectively. Panel C) presents a bar plot illustrating the substructure in the *P*. *falciparum* population at K = 2. Each vertical bar represents an individual sample and each colour represents one of the K clusters (sub-populations) defined by STRUCTURE.

In contrast to *P*. *falciparum*, no substructure was found in the *P*. *vivax* population. The neighbour-joining analysis (Fig 1A) illustrates that the *P*. *vivax* isolates exhibited low relatedness, with no distinct clustering. STRUCTURE analysis also failed to identify any evidence of sub-structure in *P*. *vivax*. In the *P*. *falciparum* population, the *delta K* method demonstrated K = 2 as the greatest likelihood, with 10.6% of isolates showing predominant ancestry (>80%) to K1 and the remaining 89.4% of isolates demonstrating predominant ancestry to K2 (Fig 1C). Neighbour-joining analysis confirmed the substructure and illustrated that neither subpopulation was the result of clonal expansion (Fig 1B). Assessment of the differentiation between K1 and K2 further confirmed local substructure (*F*_ST_ = 0.389; *p*<0.001 and *F*’_ST_ = 0.885; *p*<0.001).

Expected heterozygosity analysis revealed moderate diversity in the two *P*. *falciparum* subpopulations (^K1^*H*_E_ = 0.587 vs ^K2^*H*_E_ = 0.556; *p* = 0.818) (Table 2). However, K1 had more polyclonal infections than K2 (43.6% vs 14.4%; *p*<0.001) (Table 2). LD analysis revealed an *I*_*A*_^*S*^ of 0.041 (*p*<0.01) for K1 and 0.033 (*p*<0.01) for K2 (Table 3); the markedly lower *I*_*A*_^*S*^ scores relative to the pooled dataset suggesting admixture in the *P*. *falciparum* population.

Investigation of the demographic profiles of the K1 and K2 clusters did not find any significant difference in terms of age (*p* = 0.084), sex (*p* = 0.734), ethnicity (*p* = 0.972), or occupation (*p* = 0.096) (Table 4). Interestingly, all (100%) isolates with predominant ancestry to K1 were asymptomatic and mostly submicroscopic (71.8%) compared to 36.5% asymptomatic cases in K2 (*p* <0.001) (Table 4). No significant correlation was observed between the distance in sampling date and the proportion of alleles shared between infections (Mantel r-test, r = -0.03, *p* = 0.762). The geographical distribution of both subpopulations is shown in S3 Fig. No association was found between genetic distance and geographical distance (Mantel r-test, r = -0.03, *p* = 0.851).

**Table 4:** *P*. *falciparum* subpopulation 1 and 2 demographic data.

Subgroup (K)	Age, *n* (%)			Male, *n* (%)	Ethnicity, *n* (%)			Submicroscopic, *n* (%)	Occupation, *n* (%)					Total
	<5	5–15 y	>15		Highland	Lowland	Non-Papuan		Student	None	House wife	Miner	Farmer	
**K1**	3 (7.7)	19 (48.7)	17 (43.6)	18 (46.2)	11 (28.2)	15 (38.5)	13 (33.3)	28 (71.8)	17 (43.6)	13 (33.3)	7 (17.9)	1 (2.6)	1 (2.6)	39
**K2**	24 (7.3)	68 (20.1)	236 (72.0)	139 (42.4)	145 (44.3)	94 (28.7)	88 (26.9)	120 (36.5)	41 (12.5)	77 (23.5)	55 (16.8)	3 (0.9)	24 (7.3)	328

### The *P*. *falciparum* K1 subpopulation in Timika is related to *P*. *falciparum* parasites from other Indonesian islands

**Fig 2:**
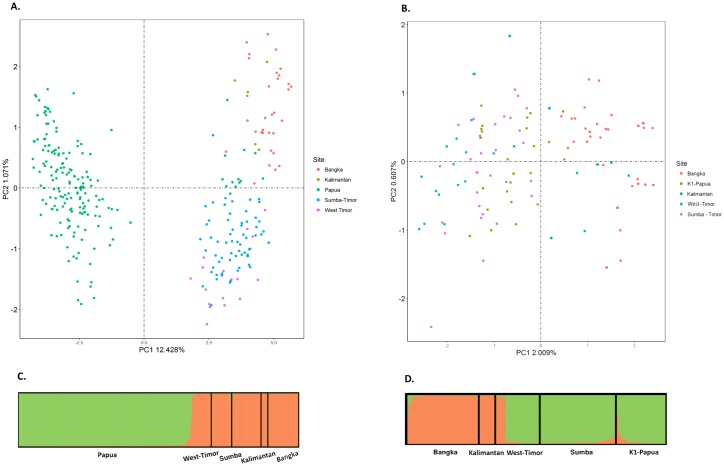
Principal coordinate analysis (PCoA) and STRUCTURE plots illustrating the genetic differentiation between *P*. *falciparum* isolates from Timika relative to Kalimantan, Bangka, Sumba and West Timor. Panels A) and B) present PCoA and STRUCTURE bar plots, respectively, illustrating the similarity between the Timika K1 subpopulation and the other four islands. The STRUCTURE bar plot presents the results for K = 2. Panels C) and D) present PCoA and STRUCTURE bar plots, respectively, for all five *P*. *falciparum* populations, with the exclusion of the Timika K2 subpopulation, illustrating the greater relatedness between K1 and Sumba relative to the other islands. The STRUCTURE bar plot presents the results for K = 2, separating K1, Sumba and West Timor in the east from Kalimantan and Bangka in the west.

To investigate the epidemiological factor(s) underlying the sub-structure in the *P*. *falciparum* population, the Timika genotypes (Papua) were compared with published data on 168 *P*. *falciparum* isolates collected from 4 surveys in other Indonesian islands (Bangka, Kalimantan, Sumba and West Timor; S4 Fig) [10]. *Delta K* evaluation of the STRUCTURE results across the 5 sites revealed that the greatest likelihood was at K = 2. Distinct separation was observed between K2 versus K1, Bangka, Kalimantan, Sumba and West Timor (Fig 2A). Principal coordinate analysis (PCoA) showed the same pattern, illustrating that K1 shared more genetic background with the non-Timika samples than with K2 (Fig 2A). Further investigation with PCoA, STRUCTURE and *F*_ST_ analysis on a restricted dataset with K1 and the other four islands showed that the K1 samples were most similar to those from Sumba and West Timor (Fig 2B; Table 5). In contrast, *P*. *vivax* differentiation was limited across the five islands (S5 Fig).

**Table 5:** Pair-wise differentiation between sites.

**Site**	**K1 Timika**	**K2 Timika**	**West Timor**	**Sumba**	**Kalimantan**	**Bangka**
**K1 Timika**	-	0.885	0.481	0.355	0.557	0.540
**K2 Timika**	0.389*	-	0.884	0.853	0.94	0.956
**West Timor**	0.226*	0.398*	-	0.241	0.719	0.653
**Sumba**	0.143*	0.343*	0.100*	-	0.555	0.561
**Kalimantan**	0.286*	0.453*	0.382*	0.248*	-	0.328
**Bangka**	0.293*	0.476*	0.361*	0.277*	0.199*	-

*F*_ST_ in lower left triangle. *F*’_ST_ in upper right triangle

*p < 1 x 10^−5^

**p = 0.011

## Discussion

This study utilised a comprehensive collection of clinical and community samples to describe the genetic microepidemiology of *P*. *falciparum* and *P*. *vivax* populations circulating in Timika, Papua Indonesia. At this high spatial resolution, we demonstrate contrasting transmission patterns of both co-existing species, and reveal admixture of two divergent *P*. *falciparum* subpopulations, one comprised of submicroscopic infections with high genetic relatedness to isolates from other Indonesian islands.

Similarly to previous reports, we found higher population and within-host diversity in co-endemic *P*. *vivax* compared to *P*. *falciparum* populations [10, 27–30]. In contrast to *P*. *falciparum*, there was no evidence of substructure in *P*. *vivax*. LD was also markedly lower in *P*. *vivax* than *P*. *falciparum*, even after accounting for admixture in the latter. These patterns are consistent with more intense and stable transmission in the local *P*. *vivax* population relative to *P*. *falciparum*. The patterns of diversity in the *P*. *falciparum* population were similar to those found in low to meso-endemic regions in Southeast Asia, but markedly less diverse than in hyper-holoendemic regions in Africa [28, 31]. Although genetic diversity estimates do not always scale linearly with endemicity in *P*. *vivax*, the patterns of *P*. *vivax* diversity in Timika were most comparable to those reported in other hypo-mesoendemic settings in South-East Asia and the Pacific [32]. Several factors may have contributed to the higher intensity and stability of *P*. *vivax* compared to *P*. *falciparum* transmission in this location, including recurrences from relapsing infections, the earlier appearance of the transmissible sexual stages in *P*. *vivax*, and the larger reservoir of submicroscopic infections in this species [3, 33].

Although the *P*. *falciparum* population exhibited several clusters of identical infections, neither species demonstrated evidence of epidemic transmission dynamics, indicating moderately stable transmission at the time of the study. The identical infections enabled assessment of shared origins of infection, revealing a small proportion (8.8% (6/68)) of isolates that were shared amongst household members, likely reflecting transmission events taking place in the vicinity of the household. However, the large majority of shared infections (70.5%) were over 1 Km apart and more likely to reflect a reservoir outside of the household. Together, the patterns of parasite relatedness, diversity and LD suggest that broad ranging interventions are still needed to further interrupt transmission of *P*. *falciparum* and *P*. *vivax* before more targeted strategies can be implemented in the area. Furthermore, although the widespread deployment of ACT has been effective in reducing the *P*. *falciparum* population, radical treatment targeting the dormant liver stages will be required to make a similar impact on *P*. *vivax* transmission.

The most notable revelation in the Timika *P*. *falciparum* population was the distinct substructure at this small spatial scale. The marked differentiation, elevated *I*_*A*_^*S*^ on pooling the K1 and K2 subpopulations, lack of evidence of epidemic transmission, together with the moderate genetic diversity within each subpopulation was indicative of admixture. However, it is unclear how sympatric *P*. *falciparum* subpopulations are genetically isolated with no sign of geographical, temporal or demographic boundaries. We contemplated three possibilities: 1) the importation of these cases, 2) the rise of a new, fitter population, and 3) an unidentified biological barrier.

Comparison of the Papuan *P*. *falciparum* genotypes with data from Bangka, Kalimantan, Sumba and West Timor revealed that the K1 isolates were more closely related to non-Papuan than Papuan isolates, and K2 was strikingly different from the rest of Indonesia. In accordance with geographic distance, the K1 sub-population demonstrated the greatest genetic similarity to the two most proximal islands, Sumba and West Timor, where *P*. *falciparum* remains hypo-mesoendemic. Owing to the mining industry and the National Transmigration Programme, there has been a great deal of human movement in Timika. Thus, it is feasible that the K1 subpopulation is the product of infections acquired outside Timika. Imported infections undermine local intervention efforts and present the threat of drug resistance introduction. Our results emphasise the need to develop geographical markers to help to differentiate between imported and local malaria cases. A recent study presented a barcode of organellar genome markers to determine the geographic origin of *P*. *falciparum* isolates [34], although this doesn’t have the resolution required to differentiate local subpopulations.

The second possibility for the unusual *P*. *falciparum* structure in Timika is the rise and spread of a fitter subpopulation in response to recent selective pressure such as the change in policy from chloroquine to ACT treatment in 2006. Parasites carrying mutations conferring chloroquine resistance may lose their survival advantage after drug pressure is removed, as has been demonstrated by the decrease in K76T *pfcrt* frequency in several African countries [35]. Assuming that the pre-ACT population was more comparable to the other Indonesian islands, the K1 subpopulation might reflect a vestigial population, whilst K2 reflects a newly emerging population. Although the recent policy change to ACT as first line-therapy was recommended for both *P*. *falciparum* and *P*. *vivax*, this treatment regimen may have had a greater impact on *P*. *falciparum*, since ACT alone has no activity against the dormant liver stages of *P*. *vivax*. However, more studies are needed to confirm this.

A further possible explanation for the sympatric co-existence of the K1 and K2 subpopulations is an unidentified biological barrier such as mutually exclusive vector preferences or differential erythrocyte binding specificities. Further investigation of the local *Anopheline* populations and their vectorial capacity for K1 and K2 is needed to address the first hypothesis. Although we did not find any evidence of association between K1 or K2 with Lowland, Highland or non-Papuan ethnicity, it is possible that a common erythrocyte polymorphism(s) defining parasite binding and invasions preference and that this transcends the different ethnic groups, and differentiates K1 and K2. Genomic analysis of these isolates might provide further insights.

Although we conducted a thorough characterisation of the subpopulations, we cannot conclusively determine the factors underlying the marked *P*. *falciparum* substructure in Timika. However, similar substructure has been observed in Cambodia, where it was postulated to support the emergence of drug resistance, highlighting a need for intense surveillance strategies [36]. It is important to highlight that the distinct substructure would not have been identified without genetic information. Furthermore, all samples from K1 were asymptomatic and mostly submicroscopic, and, therefore, would likely be missed in routine passive surveillance studies.

## Conclusions

Our study demonstrates considerable genetic heterogeneity between co-endemic species and distinct substructure within species that can be observed at small spatial scales, and may be reliant on passive as well as active case detection. These findings highlight the need for complementary new surveillance strategies to identify local transmission patterns that cannot be detected with traditional malariometric methods.

## Supporting information

S1 Fig Geographical distribution of the households at which malaria cases were detected in the cross-sectional household survey. The plot was generated using ArcGIS software on GPS coordinate data from the individuals identified with malaria parasitaemia. Each dot presents an individual case, with colour-coding by species according to PCR data.
(TIFF)

S2 Fig Geographical distribution of identical MLGs in *P*. *falciparum*. This plot was generated using ArcGIS software on the GPS coordinates for the 22 identical MLGs in the *P*. *falciparum* population. Different MLGs are distinguished with different colours and shapes. Circles, pentagons and diamonds indicate MLGs found in two, three, and more than three individuals respectively.
(TIFF)

S3 Fig Geographical distribution of the households at which *P*. *falciparum* K1 and K2 isolates were detected in the cross-sectional household survey. The plot was generated using ArcGIS software on GPS coordinate data. Each dot presents an individual *P*. *falciparum* case, with colour-coding by subpopulation as defined by STRUCTURE.
(TIFF)

S4 Fig Location of the study sites. This map is a modified version of Fig 1 presented by Noviyanti *et al*., with the addition here of a site label for Timika, Papua Indonesia [10]. The original map was generated by the Malaria Atlas Project, University of Oxford. The colour scales reflect the model-based geostatistical point estimates of the annual mean *P*. *falciparum* parasite rate in the 2–10 year age group (PfPR2–10) within the stable spatial limits of transmission in 2010. The approximate locations of the study sites described here are indicated with black stars.
(TIF)

S5 Fig Principal coordinate analysis (PCoA) between *P*. *vivax* isolates from Timika relative to Kalimantan, Bangka, Sumba and West Timor. This plot illustrates the limited differentiation between the Timika *P*. *vivax* population and the other four Indonesian islands.
(TIFF)

S1 Table Summary of the diversity and genotyping success rate for each of the *P*. *falciparum* and *P*. *vivax* markers. (DOCX)
