# Supplementary figures and images for: Genetic micro-epidemiology of malaria in Papua Indonesia: Extensive P. vivax diversity and a distinct subpopulation of asymptomatic P. falciparum infections
Source: PLoS One. 2017 May 12;12(5):e0177445. doi: 10.1371/journal.pone.0177445 (PMC5428948; doi:10.1371/journal.pone.0177445)

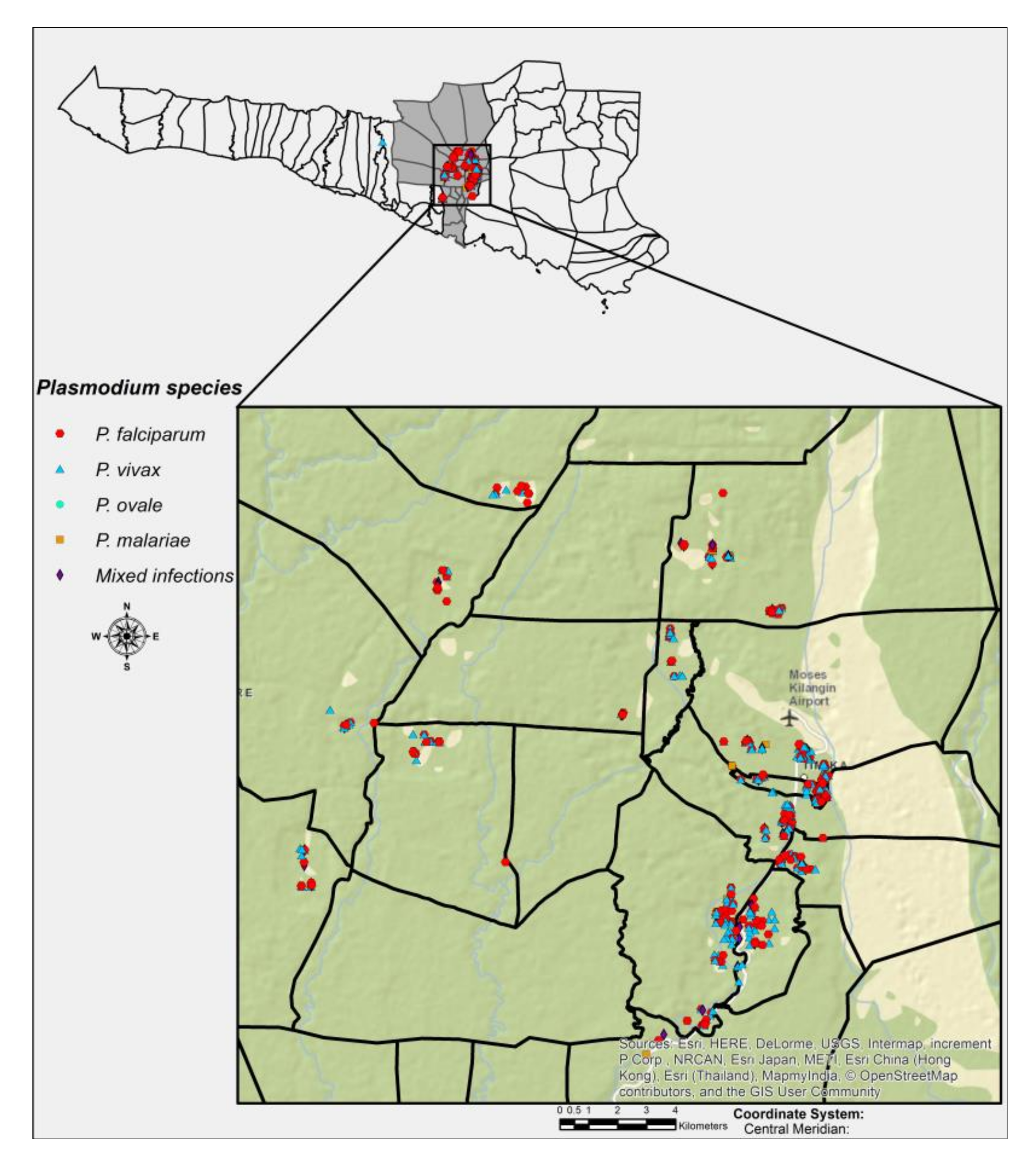

Supplement: S1 Fig — The plot was generated using ArcGIS software on GPS coordinate data from the individuals identified with malaria parasitaemia. Each dot presents an individual case, with colour-coding by species according to PCR data. (TIFF) [file pone.0177445.s001.TIFF]

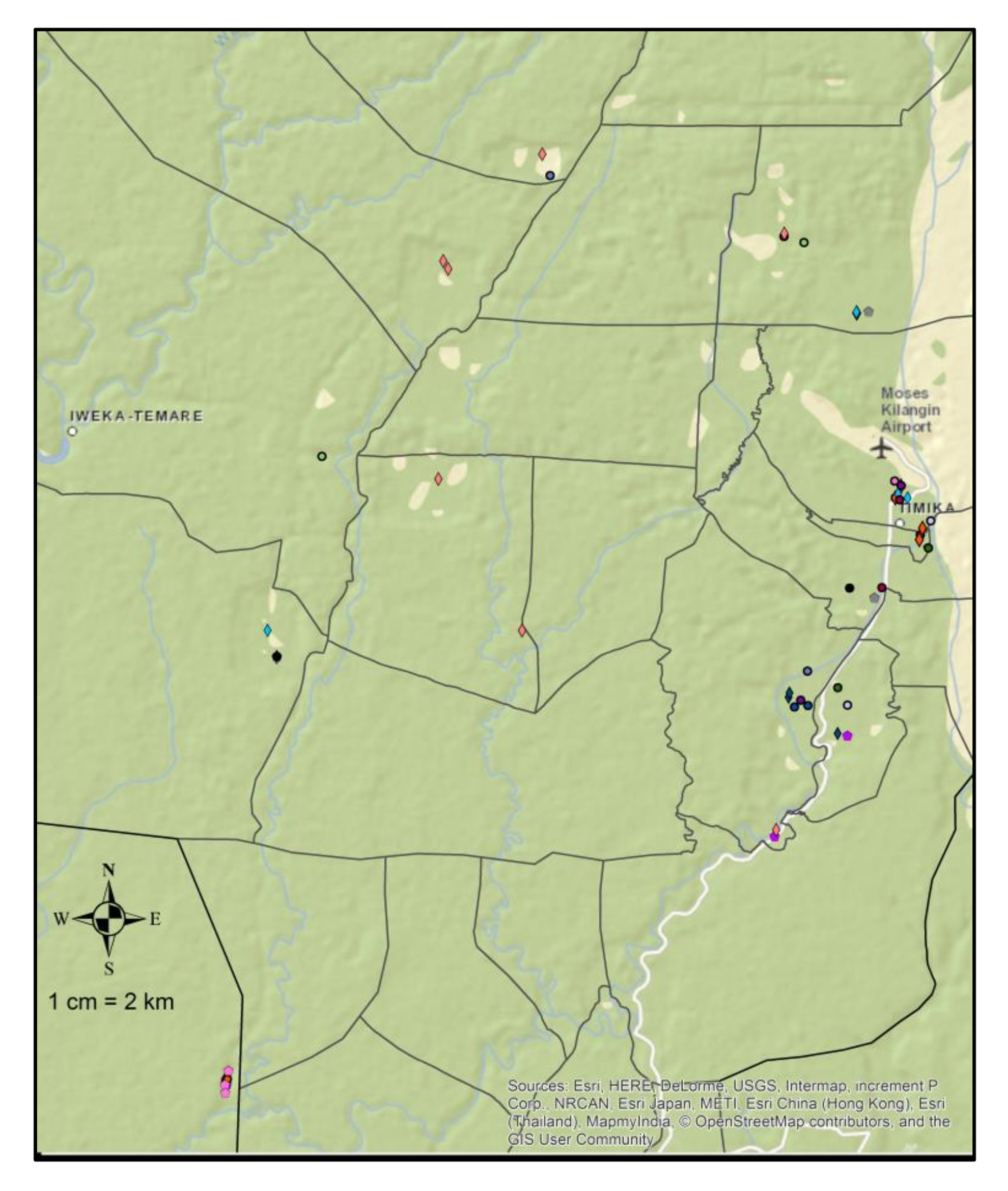

Supplement: S2 Fig — This plot was generated using ArcGIS software on the GPS coordinates for the 22 identical MLGs in the P. falciparum population. Different MLGs are distinguished with different colours and shapes. Circles, pentagons and diamonds indicate MLGs found in two, three, and more than three individuals respectively. (TIFF) [file pone.0177445.s002.TIFF]

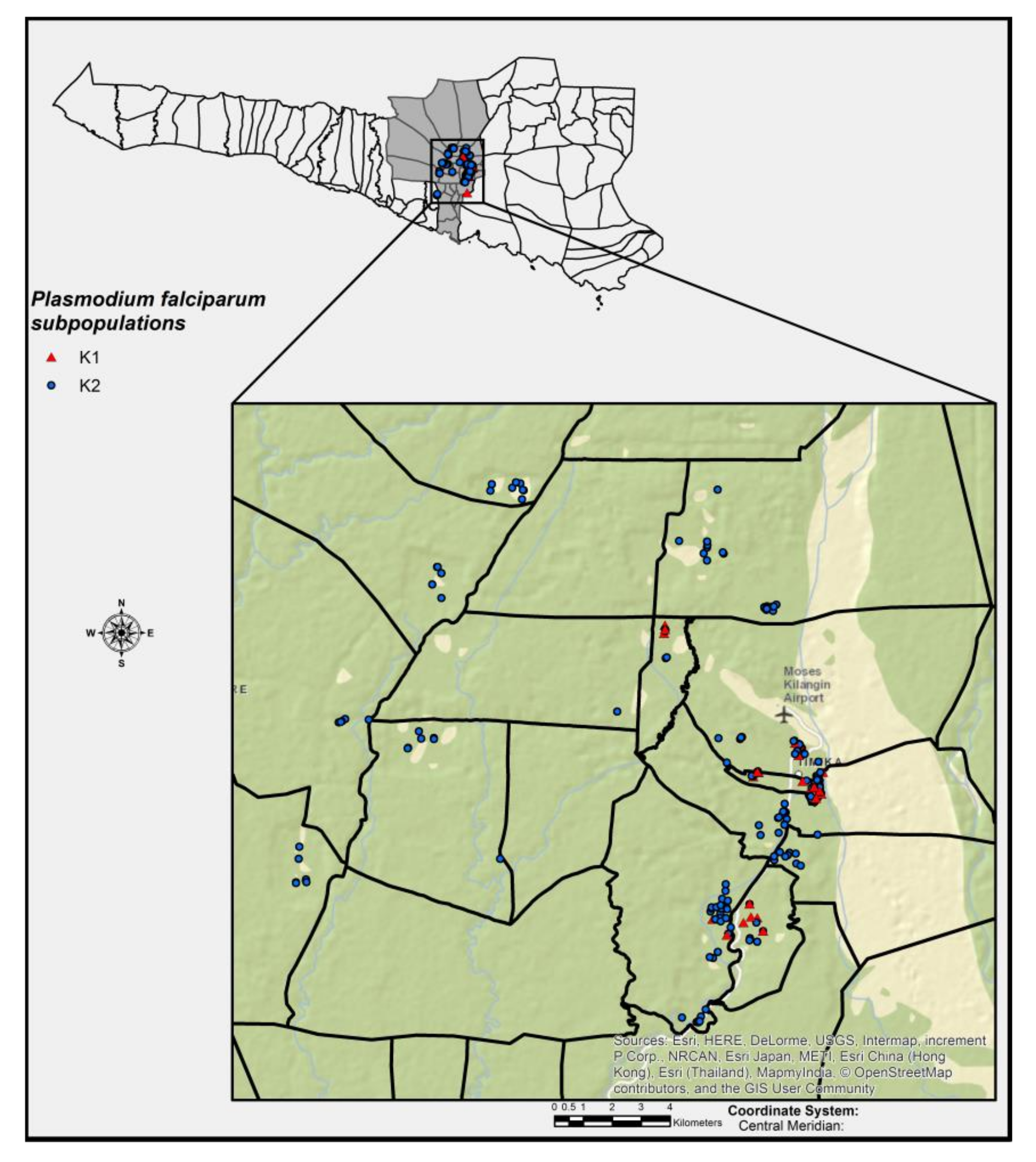

Supplement: S3 Fig — The plot was generated using ArcGIS software on GPS coordinate data. Each dot presents an individual P. falciparum case, with colour-coding by subpopulation as defined by STRUCTURE. (TIFF) [file pone.0177445.s003.TIFF]

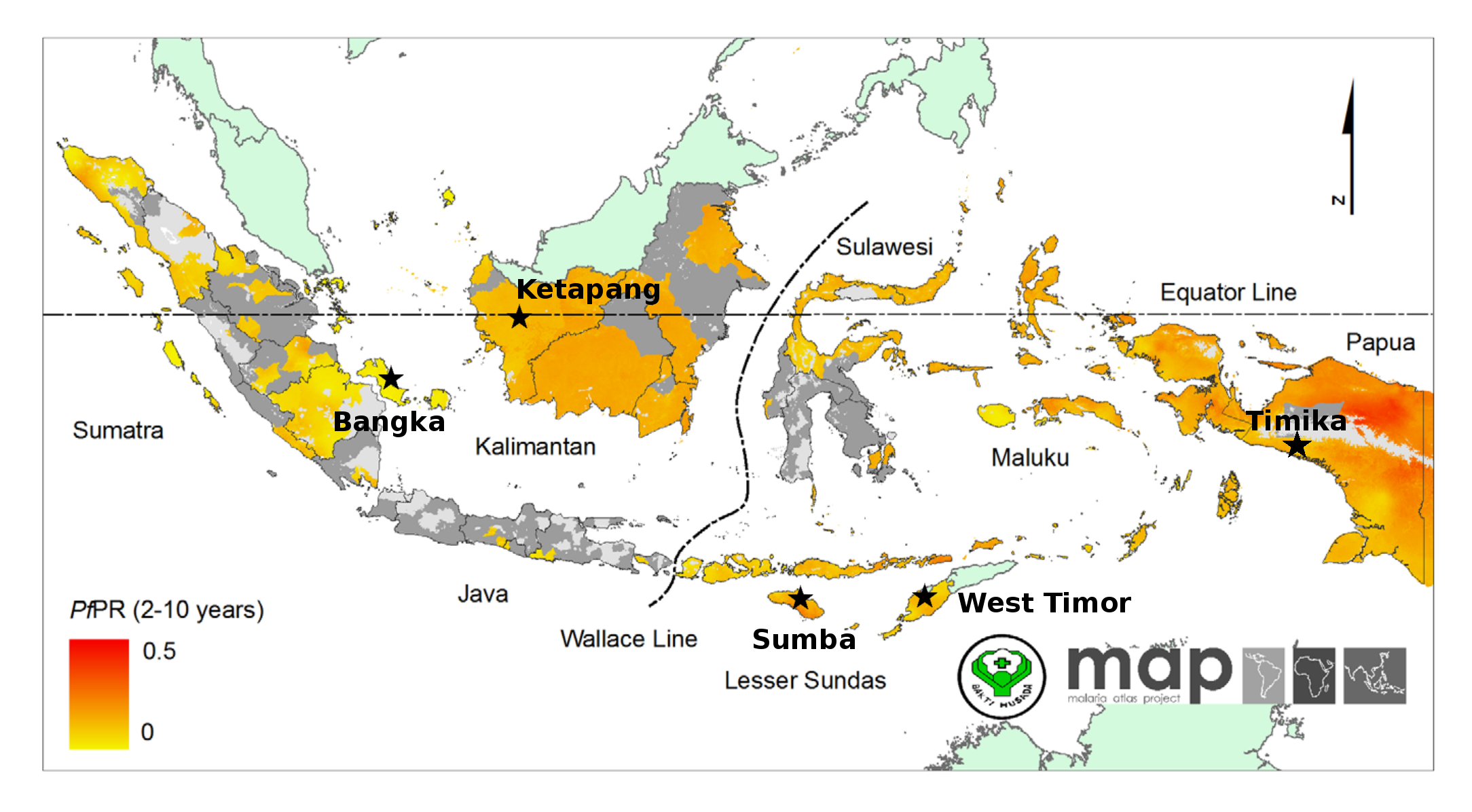

Supplement: S4 Fig — This map is a modified version of Fig 1 presented by Noviyanti et al., with the addition here of a site label for Timika, Papua Indonesia [10]. The original map was generated by the Malaria Atlas Project, University of Oxford. The colour scales reflect the model-based geostatistical point estimates of the annual mean P. falciparum parasite rate in the 2–10 year age group (PfPR2–10) within the stable spatial limits of transmission in 2010. The approximate locations of the study sites described here are indicated with black stars. (TIF) [file pone.0177445.s004.tif]

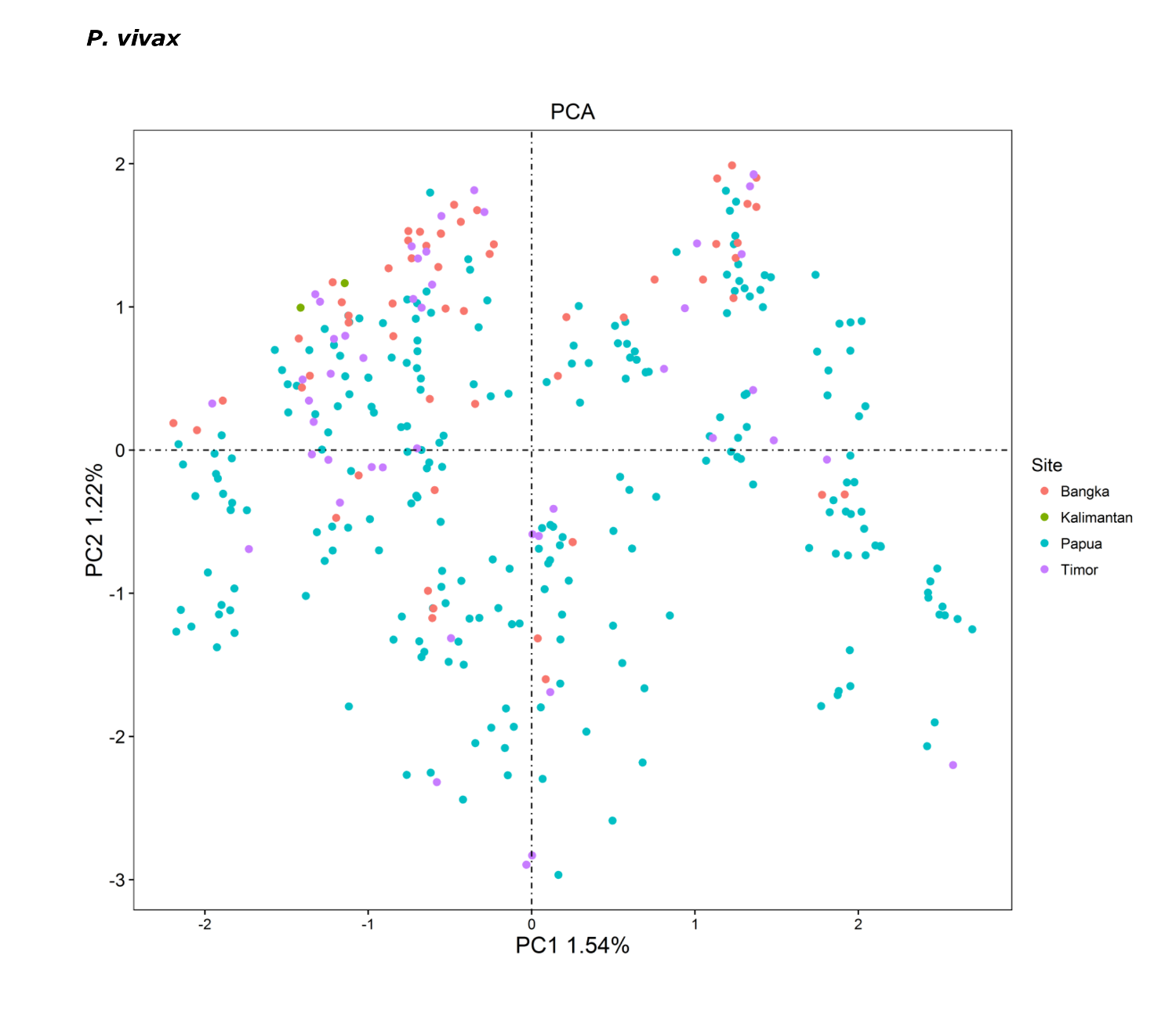

Supplement: S5 Fig — This plot illustrates the limited differentiation between the Timika P. vivax population and the other four Indonesian islands. (TIFF) [file pone.0177445.s005.tiff]
