# Supplementary material for: Genetic micro-epidemiology of malaria in Papua Indonesia: Extensive P. vivax diversity and a distinct subpopulation of asymptomatic P. falciparum infections
Source: PLoS One. 2017 May 12;12(5):e0177445. doi: 10.1371/journal.pone.0177445 (PMC5428948; doi:10.1371/journal.pone.0177445)
Supplement: S1 Table — (DOCX) [file pone.0177445.s006.docx]

**S1 Table. Summary of the diversity and genotyping success rate for each of the *P. falciparum* and *P. vivax* markers.**

| **Species** | **Marker** | ***H*_E_** | **Failure, *n* (%)** |
| --- | --- | --- | --- |
| ***P. vivax*** | *MS12* | 0.741 | 28 (8.1) |
|  | *pv3.27* | 0.879 | 32 (9.3) |
|  | *msp1f3* | 0.808 | 36 (10.5) |
|  | *MS10* | 0.899 | 21 (6.1) |
|  | *MS5* | 0.849 | 16 (4.7) |
|  | *MS1* | 0.77 | 23 (6.7) |
|  | *MS16* | 0.941 | 33 (9.6) |
|  | *MS20* | 0.891 | 53 (15.4) |
| ***P. falciparum*** | *Poly-alpha* | 0.717 | 12 (3.3) |
|  | *TA42* | 0.315 | 76 (20.6) |
|  | *TA81* | 0.82 | 40 (10.8) |
|  | *TA87* | 0.672 | 13 (3.5) |
|  | *ARAII* | 0.805 | 12 (3.3) |
|  | *PfPK2* | 0.722 | 19 (5.1) |
|  | *TA60* | 0.724 | 4 (1.1) |
|  | *TA1* | 0.679 | 62 (16.8) |
|  | *TA109* | 0.176 | 10 (2.7) |
